# How Emotional Intelligence Influences Students’ Life Satisfaction During University Lockdown: The Chain Mediating Effect of Interpersonal Competence and Anxiety

**DOI:** 10.3390/bs14111059

**Published:** 2024-11-07

**Authors:** Yuyang Zhao, Biao Sang, Cody Ding

**Affiliations:** 1 Department of Social Work, School of Sociology and Political Science, Shanghai University, Shanghai 200444, China; 2 Lab for Educational Big Data and Policymaking, Shanghai Academy of Educational Sciences, Shanghai 200032, China; 3 Department of Education Science and Professional Programs, University of Missouri-Saint Louis, St. Louis, MO 63121, USA

**Keywords:** COVID-19 pandemic, adolescent, life satisfaction, trait emotional intelligence, interpersonal competence, anxiety

## Abstract

Students’ life satisfaction is an essential reflection of their well-being, particularly during challenging times. The COVID-19 pandemic, a global catastrophe, has had an immeasurably negative impact on individuals’ daily lives. It has also provided an opportunity to investigate the factors contributing to students’ life satisfaction during such difficult times. Considering the unique characteristics of these university students, the current study examines the impact of emotional intelligence (EI) on students’ life satisfaction, as well as the direct and sequential mediating effects of interpersonal competence and anxiety on this relationship. A total of 297 university students in Shanghai, China, participated in the study. All participants were recruited online during the early phase of the COVID-19 pandemic in 2022. Four well-established questionnaires were utilized to assess students’ trait emotional intelligence, interpersonal competence, anxiety, and life satisfaction. The findings indicate that interpersonal competence significantly mediates the relationship between EI and life satisfaction. Although anxiety does not significantly mediate this relationship, it plays a notable role in the sequential mediating effect involving interpersonal competence and anxiety. The study reveals that nurturing students’ EI can significantly enhance their interpersonal competence, which can reduce anxiety and ultimately increase their self-reported life satisfaction.

## 1. Introduction

Satisfaction with life is a widely recognized global indicator for self-reported well-being, encompassing various aspects of an individual’s life [1,2,3], including social behaviors, education, and physical and mental health, across different cultures. Life satisfaction has come to be recognized as a distinguished indicator for individuals’ daily functioning [4,5], especially in psychosocial functions [6,7]. Individuals with higher levels of life satisfaction tend to exhibit more prosocial behaviors [8] and are less prone to antisocial behaviors [9], especially in children and adolescents [10,11]. Therefore, life satisfaction emerges as a pivotal determinant of children’s social behaviors. Among all the factors that contribute to the sense of life satisfaction, resilience, social support, social relationships, and emotion regulation have been found to predict life satisfaction positively [12,13,14]; whereas, stress, anxiety, and depression have been shown to be negatively associated with life satisfaction [15,16], and are often accompanied by negative life experiences or events [17,18].

Undoubtedly, the coronavirus disease 2019 (COVID-19) emerged as one of the most formidable calamities to afflict modern human society [19], reshaping individuals’ lives in unprecedented ways. In response to the gripping threat of COVID-19, numerous nations initiated sweeping lockdown measures, both nationally and regionally, to mitigate the virus’s spread. These measures encompass a spectrum of stringent restrictions to curtail human movement and interaction. From the suspension of international and domestic travel to the limitation of social gatherings and physical engagements, the impact has been profound. Moreover, access to vital public services, including healthcare and education, has been reconfigured, reflecting the far-reaching implications of this global health crisis. The imposition of international, national, and regional quarantines has catalyzed a profound transformation in the conventional educational paradigm, transitioning from the traditional stationary learning environment to the dynamic realm of remote learning [20,21]. Passive life shifts, exemplified by the disruptions caused by COVID-19, coupled with the transition to new learning modalities, have been linked to a cascade of adverse psychological repercussions. These include elevated stress and anxiety levels, a pervasive sense of depression [22,23], and a discernible decline in life satisfaction [24,25]. Moreover, outbreaks of infectious diseases like COVID-19 can trigger profound emotional distress and elicit a spectrum of negative emotional responses [26], which can be effectively managed or protected by trait emotional intelligence (EI) [27]. During the COVID-19 pandemic, a vast array of universities across China implemented lockdown protocols, asking students to remain within the confines of their campuses or dormitories. Despite this, few studies examined the effect of students’ EI on their life satisfaction during the campus lockdown. Moreover, students who were asked to reside on campus encountered the challenge of an escalating frequency and intensity of interpersonal engagements and negative emotions such as anxiety. This situation markedly intensified the frequency of student interactions, offering a unique opportunity to closely scrutinize the impact of interpersonal engagement on anxiety levels. Therefore, the current study investigated the relationship between university students’ trait EI and life satisfaction during the university lockdown, as well as the mediating role of interpersonal competence and anxiety in such a relationship.

### 1.1. Emotional Intelligence and Life Satisfaction

Research has consistently demonstrated that EI is a critical predictor of numerous life outcomes, including personal success and general well-being [28,29,30]. Regardless of the specific type of EI [31] (i.e., ability and trait EI), a substantial body of empirical studies have consistently demonstrated the positive relationship between EI and life satisfaction across different types of samples, such as adolescents or young adults [32,33], educators [34], and healthcare professionals [35]. Beyond the correlational relationship, a recent longitudinal study revealed that trait EI is a stable personality trait and exerts a long-lasting directional influence on relationship satisfaction in later life [36], which was often perceived as a pivotal component of overall life satisfaction [37]. Yet, the stability of this influence during adverse historical events, such as pandemics, remains a subject of inquiry.

In addition to the directional effect of trait EI on life satisfaction, researchers also investigate different mediators in the relationship between trait EI and life satisfaction [38,39,40] in everyday situations. Furthermore, researchers found that EI could help individuals complete emotional decision-making tasks during emotionally difficult situations [41], and the benefit of training EI on adolescents’ well-being was found in both clinical and community preventive levels [42,43]. Compared to ability EI, trait EI is more pertinent due to its emphasis on the inherent tendencies and perceptions related to emotions [29], which align closely with the exploratory nature of the current research.

Even though studies indicated that students’ trait EI and life satisfaction tend to be lower during the pandemic [44], few studies investigated the relationship between trait EI and individuals’ general well-being, such as life satisfaction, during obstacles or adversity. Expanding upon the well-established directional and positive impacts of trait EI on life satisfaction within everyday contexts, this research endeavored to scrutinize the resilience and adaptability of this relationship amidst challenging historical events, such as the COVID-19 pandemic, and adverse situations like university lockdowns. The current study hypothesizes that:

**Hypothesis** **1:** *Students* *’ trait emotional intelligence has a positive effect on their life satisfaction.*

### 1.2. Emotional Intelligence, Interpersonal Competence, and Life Satisfaction

In addition to the importance of EI, interpersonal relationships are a pivotal aspect throughout an individual’s lifespan, particularly during formative years [45]. Although some studies have argued that there was a critical difference in the nature and significance of friendship between children and adolescents [46], most researchers agree that interpersonal relationships have a positive impact on early human development [47]. Interpersonal relationships, as an important foundation of social support, are critical resources that individuals can utilize to overcome obstacles [48]. Previous studies have found that children’s interpersonal and intrapersonal skills significantly declined during the COVID-19 pandemic, potentially attributable to the concurrent decline in their EI [44]. Although the relationship between EI and interpersonal competence was not examined during the COVID-19 pandemic, previous studies have established a correlational relationship among university students [49] and employees [50]. Moreover, research has demonstrated a positive impact of trait EI on interpersonal competence, particularly in adolescents [51]. Specifically, trait EI has been found to have a positive effect on appropriate social skills and a negative effect on inappropriate social skills [37]. This suggests that individuals with higher trait EI may be better equipped to navigate social interactions effectively, even in challenging circumstances such as a pandemic or university lockdowns.

Furthermore, satisfactory social connections are a robust contributor to individuals’ well-being, including satisfaction with life. Even though few studies have examined the effect of interpersonal competence on life satisfaction [52], most previous studies have emphasized the importance of interpersonal relations for life satisfaction [53,54]. Interpersonal relations were found to be outcomes of interpersonal competence or skills in children and adolescents [55,56]. Social support studies further indicated that individuals’ interpersonal competence could be a protective factor against stressful life events [57]. During periods of university lockdown, university students were asked to remain on campus, which significantly amplifies their interpersonal interactions within a relatively confined social network. This increased density of social contact may exacerbate issues related to interpersonal relationships. Consequently, students with higher levels of interpersonal competence are likely to experience reduced negative emotions and increased well-being [58], such as life satisfaction.

In this study, interpersonal competence is posited as the primary mediator due to its potential to be a significant obstacle that university students encounter during university lockdowns. By examining the impact of students’ interpersonal competence, this research endeavors to elucidate how EI influences their interpersonal competence, potentially leading to more positive consequences, such as heightened life satisfaction. Consequently, interpersonal competence is identified as a pivotal mediating factor in the pathway from EI to life satisfaction, exerting a direct effect on individuals’ satisfaction with life. The current study hypothesizes that:

**Hypothesis** **2a:** *Students* *’ trait emotional intelligence has a positive effect on their interpersonal competence;*

**Hypothesis** **2b:** *Students* *’ interpersonal competence has a positive effect on their life satisfaction;*

**Hypothesis** **2c:** *Students* *’ interpersonal competence serves as a mediator in the relationship between emotional intelligence and life satisfaction.*

### 1.3. Emotional Intelligence, Anxiety, and Life Satisfaction

The COVID-19 pandemic has been associated with significant stress for individuals, particularly affecting adolescents [59]. Anxiety levels among adolescents have been reported to be a critical issue that requires attention during the pandemic [60]. More specifically, previous systematic reviews and meta-analyses have reported an anxiety prevalence of 31.9% in the general population [61], and a notably higher rate of 41% among university students during the COVID-19 pandemic [62]. Compared to pre-pandemic data, adolescents exhibited a significant increase in general anxiety symptoms during the pandemic, which can be attributed to pandemic-related restrictions and school closures [63]. It is important to identify a protector for individuals during challenging times like the pandemic. EI involves the ability to recognize, understand, and manage one’s own emotions and those of others, which can be particularly beneficial during times of such crisis. Previous empirical studies have indicated a negative relationship between EI and general anxiety [64,65,66] in everyday situations. In addition, trait EI was found to have a positive effect on different types of stress-related coping strategies [67] during the COVID-19 measures. Thus, individuals with higher EI are more likely to employ positive coping strategies, which can reduce feelings of anxiety [68].

Moreover, during the pandemic, adolescents’ anxiety could arise from various aspects, such as fear of infection, online learning, and social isolation. This anxiety might either interfere with student’s ability to experience satisfaction in different areas of life or disrupt students’ cognitive processes. Such disruption makes it difficult for them to focus on and appreciate the positive aspects of their life, thus contributing to lower life satisfaction. It is evidenced that Vietnamese university students’ anxiety negatively impacted their life satisfaction during the COVID-19 pandemic [69]; such directional impact was also found in non-pandemic periods among diverse cultures [70,71,72]. More importantly, general life satisfaction typically declines during adolescence [73], potentially due to stressful events that are more likely to trigger maladaptive internal responses, such as anxiety [74]. Thus, stressful events, such as the COVID-19 pandemic, have the potential to elevate anxiety levels among university students, which in turn can adversely affect their life satisfaction.

In this study, anxiety is posited as the second mediator because it reflects the cumulative impact of trait EI as well as its high prevalence and significant influence during the COVID-19 pandemic. Trait EI, the ability to understand and regulate emotions, is regarded as a pivotal strategy for combating anxiety [27,75] and increasing life satisfaction [40,76]. Therefore, the current study investigated the mediating effect of anxiety on the relationship between trait EI and students’ life satisfaction during university lockdown due to the pandemic. The current study hypothesizes that:

**Hypothesis** **3a:** *Students* *’ trait emotional intelligence has a negative effect on their anxiety;*

**Hypothesis** **3b:** *Students* *’ anxiety has a negative effect on their life satisfaction;*

**Hypothesis** **3c:** *Students* *’ anxiety serves as a mediator in the relationship between emotional intelligence and life satisfaction.*

### 1.4. Emotional Intelligence, Interpersonal Competence, Anxiety, and Life Satisfaction

Interpersonal relationships are fundamental to an individual’s emotional state and overall well-being, especially during a university lockdown. The impact of interpersonal skills on anxiety has been evidenced [77]. Individuals with a higher degree of interpersonal competence often demonstrated reduced anxiety levels [78]. A recent study further found that the increased time spent in a confined social network on campus during lockdowns may intensify interpersonal relationship issues (e.g., isolation from peers and integration among peers), potentially leading to higher negative emotions and decreased well-being for students [79].

Many factors might contribute to interpersonal relationship issues, besides the fear of being negatively evaluated by others and the avoidance of such fear being activated [80], individuals might also experience anxiety when they are obliged to remain in an interpersonal interaction. Studies have shown that university students often experience challenges in maintaining harmonious relationships with their peers and roommates in everyday life [81,82]. Accordingly, individuals who perceive themselves as more capable of handling interpersonal interactions may be less likely to experience anxiety in such situations. Moreover, previous studies have found an indirect effect of interpersonal competence on life satisfaction, with dating anxiety serving as a mediator [53]. Therefore, this study proposes that university students’ life satisfaction during the COVID-19 pandemic may be significantly influenced by their level of anxiety, which can, in turn, be affected by their interpersonal competence. Additionally, it is well-established in prior research that individuals’ interpersonal competence is largely determined by their trait EI [50,51]. Accordingly, the current study hypothesizes that:

**Hypothesis** **4:** *Students* *’ interpersonal competence and anxiety serve as serial mediators in the relationship between trait emotional intelligence and life satisfaction.*

### 1.5. The Present Study

**Figure 1:**
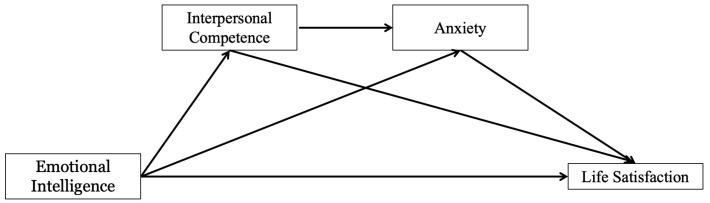
Serial mediating effects of interpersonal competence and anxiety in links from emotional intelligence to life satisfaction.

Based on the above discussion, this study proposes a chain mediating model (Figure 1) that delves into the nexus between trait EI and life satisfaction. While previous research has established a link between trait EI and life satisfaction, there is a scarcity of studies that have examined the potential of trait EI to enhance life satisfaction by strengthening interpersonal competence and reducing anxiety within a single model, especially during significant life events such as the COVID-19 pandemic. This study aims to elucidate the impact of trait EI on adolescents’ perception of life satisfaction and to reveal the mediating effects of interpersonal competence and anxiety. The findings seek to offer theoretical insights into the development of adolescents’ life satisfaction.

## 2. Materials and Methods

### 2.1. Participants

All participants were recruited after obtaining approval from the Ethics Committee of Shanghai University following the general rules of the Declaration of Helsinki and Measures for Ethical Review of Biomedical Research Involving Humans, Ministry of Health, China.

This study was part of interventional research aimed at enhancing peer support among university students during the COVID-19 pandemic’s university lockdown. After deleting and testing the missing data and outliers, the participants in the current study included 297 university students from a first-class university (in 2017, China lunched a national initiative in higher education called the “Double-First Initiative” to promote the development of universities in China) in Shanghai China. All data were collected online via an online research platform similar to SurveyMonkey or Qualtrics, called WenJuanXing. Of the participants, 76.4% (227) were female. The mean age of the sample was 21.39 (SD = 3.31), 90.6% (269) of the participants are of the Han ethnicity, and 68% (202) of the participants were undergraduate students.

### 2.2. Measures

#### 2.2.1. Emotional Intelligence

Students’ trait EI was measured by a 33-item self-reported EI test (SREIT), which was developed by Schutte et al. [83], based on Salovey and Mayer’s [84] theoretical framework of EI. The SREIT comprises three factors: appraisal and expression of emotion (13 items), regulation of emotion (10 items), and utilization of emotion (10 items). Schutte et al. [83] reported internal consistency reliability (α = 0.87) and test–retest reliability (α = 0.78), and the measurements were validated in China [29]. The internal reliability in the current study is 0.92. For other relevant descriptive statistics, see Table 1. Students indicated how well each statement describes them using a 4-point Likert scale (1 = strongly disagree to 4 = strongly agree) because of the mid-point effect or social desirability bias [85]. Example questions for appraisal and expression of emotion factor include “I like to share my emotions with others”, “other people find it easy to confide in me” for the regulation of emotion factor, and “I expect good things to happen” for the utilization of emotion factor. Items 5 and 28 were reverse coded. A mean score was computed to represent the level of EI.

#### 2.2.2. Anxiety

Students’ anxiety was measured by the symptom checklist 90 revised (SCL-90R) anxiety subscale (10 items), which was developed by Derogatis and Cleary [86] with an internal consistency reliability of 0.85. The internal reliability in the current study is 0.93. For other relevant descriptive statistics, see Table 1. Students indicated how well each statement described them during the last week using a 5-point Likert scale (1 = never to 5 = extremely). Example questions are “I feel lonely” and “cry easily”. A mean score was computed to represent the level of anxiety.

#### 2.2.3. Interpersonal Competence

Students’ interpersonal competence was measured by a 40-item interpersonal competence questionnaire (ICQ), which was developed by Buhrmester et al. [87] with an internal consistency reliability of 0.88. The ICQ comprises five domains: initiation (8 items, α = 0.88), negative assertion (8 items, α = 0.90), disclosure (8 items, α = 0.82), emotion support (8 items, α = 0.90), and conflict management (8 items, α = 0.84). The ICQ was validated in the context of Chinese university students and reported a reliability of 0.82 [88]. The internal reliability for the whole scale in the current study is 0.96. For other relevant descriptive statistics, see Table 1. Students indicated their level of competence and comfort in handling the situation (1 = “I’m poor at this; I’d feel so uncomfortable and unable to handle this situation, I’d avoid it if possible”; 2 = “I’m only fair at this; I’d feel uncomfortable and would have lots of difficulty handling this situation”; 3 = “I’m OK at this; I’d feel somewhat uncomfortable and have some difficulty handling this situation”; 4 = “I’m good at this; I’d feel quite comfortable and able to handle this situation”; 5 = “I’m extremely good at this; I’d feel very comfortable and could handle this situation very well”). Example questions for initiation domains include “Introducing yourself to someone you might like to get to know (or date)” and “Turning down a request by a companion that is unreasonable” for the negative assertion domain; “Being a good and sensitive listener for a companion who is upset” for the emotional support domain; “Letting a new companion get to know the ‘real you’” for the disclosure domain; and “I expect good things to happen” for the utilization of emotion factor. A mean score was computed to represent the level of interpersonal competence.

#### 2.2.4. Life Satisfaction

Students’ life satisfaction was assessed by the satisfaction with life scale (SWLS), which was developed by Diener et al. [89]. The SWLS is a short five-item general life satisfaction scale wherein participants indicate to what extent they agree with certain statements using a seven-point Likert scale (1 = strongly disagree to 7 = strongly agree). The internal reliability in the current study is 0.88. For other relevant descriptive statistics see Table 1. A six-point scale was used in the current study, and the mid-point was deleted to avoid the mid-point effect or social desirability bias [85]. Example SWLS questions include “In most ways, my life is close to my ideal” and “If I could live my life over, I would change almost nothing”. A mean score was computed to represent the level of life satisfaction.

### 2.3. Procedure

All participants were recruited during the university lockdown due to COVID-19 in March 2022, which was before the time of the city lockdown in Shanghai. During this period, all students were asked to stay on campus and take online classes in the dormitory. Participants were enrolled via an online survey platform, targeting individuals pursuing academic degrees in social work, encompassing both undergraduate and graduate levels. A snowballing method was employed. A digital promotional poster, featuring a QR code and an embedded hyperlink, was disseminated online to all cohorts of students who had expressed a voluntary interest in participating in the survey. Students who were interested in this study scanned the QR code and were directed to an online research platform, similar to Survey Monkey, to fill out the consent form before taking the survey. The survey takes approximately 10 min to finish. No rewards were included. All participants were informed that the results would be anonymous and there was no right or wrong answer to the question before they took the survey. Basic demographic information (i.e., gender, age, grade, and race) was collected.

### 2.4. Data Analysis

In the current study, SPSS software (version 26.0) was employed. For the purpose of the current study, the common method bias, data normality, scale reliability, descriptive statistics, correlation analysis, and multicollinearity tests were first performed. Then, the hypothesis was examined using the PROCESS macro by Hayes (model 6) [90].

## 3. Results

### 3.1. Common Method Bias and the Normality of the Data

Moreover, as Kock et al. [91] suggested, anonymous response collection and reverse-coded items were employed to decrease the effect of potential common method bias. Harman’s single-factor test was performed to test the common method bias. The findings from the initial exploratory factor analysis, conducted with the unrotated solution on all items within each measurement, revealed that 17 factors had eigenvalues greater than one. The variance attributed to the greatest factor was 28.43% (below 50%), suggesting that common method bias was not detected in the current research.

Quantile–quantile (Q-Q) plots were utilized to graphically assess the normality of the variable distributions in the study. The results from the Q-Q plots for all assessments showed that most of the data points align with diagonal lines in these plots indicating the data normality of the current study.

### 3.2. Descriptive Statistics and Correlation Analysis

The mean value, standard deviation, and Pearson correlation matrix are shown in Table 1. The correlational analysis showed that EI was significantly and positively related to life satisfaction and interpersonal competence, but significantly and negatively related to anxiety. Life satisfaction was significantly and positively related to interpersonal competence, but significantly and negatively related to anxiety. Anxiety was significantly and negatively related to interpersonal competence. In addition, gender was significantly and negatively related to interpersonal competence.

### 3.3. Chain Mediation Model Test

The multicollinearity test was performed before the mediation analysis, as suggested by O’ Brien [92]; the Variance Inflation Factor (VIF) values of all variables are less than the critical value of 3, indicating that there was no collinearity in the model. Additionally, to reduce the statistical error of the chain mediation model, gender and age were used as control variables for the following analysis. The chain mediation analysis was performed following the instructions of Hayes (2017) [90]. Standardized coefficients were calculated.

**Figure 2:**
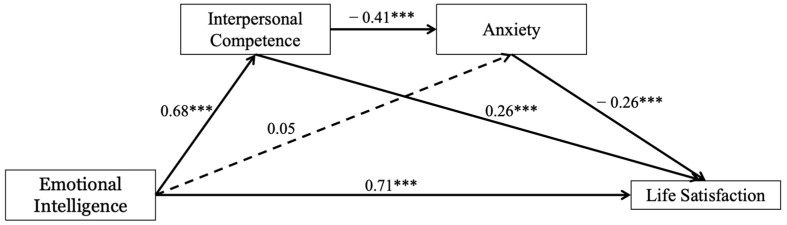
The chain mediating model of emotional intelligence, interpersonal competence, anxiety, and life satisfaction. Note: standardized coefficients were presented as the direct effect between variables; solid lines refer to significant impact, and dotted lines refer to insignificant impact; *** *p* < 0.001.

As shown in Table 2, trait EI had a significantly positive and direct effect on interpersonal competence and life satisfaction, but did not significantly predict anxiety. Interpersonal competences had a significantly negative and direct effect on anxiety and a positive and direct effect on life satisfaction. Anxiety had a significantly negative and direct effect on life satisfaction (Figure 2).

Based on the results of 5000 bootstrap samples, the mediating effect of interpersonal competence and anxiety was tested by Hayes’ PROCESS macro. As shown in Table 2 and Table 3, interpersonal competence had a significantly positive and direct effect on life satisfaction (*β* = 0.26, *SE* = 0.22, *p* < 0.001). The mediating effect test showed a mediating effect value of 0.18 and a 95% confidence interval of [0.07, 0.31] not including 0, the proportion of variance in the total effects explained by this mediation was 38.30%. The results indicated that interpersonal competence positively impacts life satisfaction, and partially mediates the relationship between EI and life satisfaction.

As shown in Table 2 and Table 3, EI had no significant and direct effect on anxiety (*β* = 0.05, *SE* = 0.17, *p* = 0.54). Anxiety had a significantly negative and direct effect on life satisfaction (*β* = −0.26, *SE* = 0.07, *p* < 0.001). The mediating effect test showed a mediating effect value of −0.01 and a 95% confidence interval of [−0.07, 0.05] including 0, the proportion of variance in the total effects explained by this mediation was 2.13%. The results indicated that anxiety did not mediate the relationship between trait EI and life satisfaction.

As shown in Table 2 and Table 3, interpersonal competence has a significant negative and direct effect on anxiety (*β* = −0.41, *SE* = 0.09, *p* < 0.001). The mediating effect test showed a mediating effect value of 0.07 and a 95% confidence interval of [0.03, 0.12], the proportion of variance in the total effects explained by this mediation was 14.89%. The results indicate that interpersonal competence and anxiety played a significant chain mediating effect on the relationship between trait EI and life satisfaction.

## 4. Discussion

This research stands as a pioneering effort to examine the interconnections between EI, interpersonal competence, anxiety, and life satisfaction within the context of university students during the COVID-19 pandemic. The results from the current study supported the mediating effect of interpersonal competence as well as the chain mediating effect of interpersonal competence and anxiety on the relationship between EI and life satisfaction.

### 4.1. The Direct Effect of Emotional Intelligence on Life Satisfaction

The present study has revealed that EI exerts a significant and positive influence on life satisfaction, thereby corroborating Hypothesis 1 and aligning with the findings of previous research [28,29,30,31,32,33,34,35,36,37,38,39,40]. Additionally, this study offers additional empirical validation of this relationship during a critical period of life transition, characterized by the period when students were mandated to remain on campus or in dormitories as a result of the COVID-19 pandemic. Individuals with higher EI are not only more satisfied with life during ordinary circumstances [36,76], but also appear to be more resilient when faced with challenging situations. When individuals encounter challenging situations, they typically report a decreased level of life satisfaction [24,25] and negative emotional reactions [63], which can be mitigated by EI [41]. According to the findings of the current study, enhancing EI might be one possible solution to boost individuals’ life satisfaction. Therefore, it might be crucial to foster individuals’ EI to equip them with the ability to navigate life’s difficulties more effectively, especially during the life uncertainty period.

### 4.2. The Mediating Role of Interpersonal Competence and Anxiety

The current study confirmed the positive impact of EI on interpersonal competence and the positive influence of interpersonal competence on life satisfaction, which is consistent with previous studies [47,57]. Additionally, the results of the current study further underscored the mediating role of interpersonal competence in the relationship between EI and life satisfaction. Thus, EI not only directly affects students’ life satisfaction during the COVID-19 pandemic, but also through the mediating effect of interpersonal competence. One possible explanation for the significant mediating role of interpersonal competence is attributed to the shift in social interaction dynamics during the university lockdown, marked by a change in the intensity and frequency of interactions among peers. Under certain conditions, students might find themselves in situations of “forced peer integration” where they have to interact with peers or roommates they did not particularly like or in which they are isolated from peers or roommates [79]. In these contexts, the student’s capacity to appraise, express, regulate, and utilize their emotions might become increasingly vital. During the university lockdown, students with higher EI might be capable of developing stronger interpersonal competence and fostering more harmonious and satisfied relationships with peers, which was one of the major parts of their daily life on campus. As a result, they feel more satisfied with life. Building upon the well-established study conducted by Schutte et al. [50] on EI, interpersonal relations, and satisfaction in relations, the present study further elucidated the positive influence of EI on general life satisfaction through the mediating effect of interpersonal competence, particularly in the context of challenged social interactions.

Meanwhile, in contradiction with previous studies [62,63,64,65,66,67], the current study did not identify a predictive role of EI on anxiety, nor did it evidence the mediating effect of anxiety on the relationship between EI and life satisfaction. One potential explanation for the non-significant relationship might be due to the unique social context that the students find themselves in. During the COVID-19 pandemic, the social environment and interaction patterns were significantly altered, which could have influenced the way EI relates to anxiety and life satisfaction. For example, emotional issues may not be the sole contributor to anxiety during the COVID-19 pandemic. A spectrum of clinical manifestations [93], neurological symptoms [94], and cognitive dysfunctions [95] could also substantially fuel students’ anxiety, and these concerns may not be fully addressed by EI. Moreover, researchers recently found that emotional clarity and repair had a mediating effect between social interaction anxiety and satisfaction with life [96]. Thus, the relationship between EI and anxiety appears to be intricate and interactive. Although the correlational link between EI and different types of anxiety is well established [64,65,66], further research is necessary to elucidate the directional nature of this relationship. Therefore, considering the social context and the direction of the relationship is crucial for a comprehensive examination of the relationship between EI and anxiety.

### 4.3. The Chain Mediating Role of Interpersonal Competence and Anxiety

The results of the current study supported Hypothesis 4, suggesting that interpersonal competence and anxiety sequentially mediate the relationship between students’ trait EI and life satisfaction during the university lockdown. In other words, amidst socially challenging scenarios such as the COVID-19 pandemic, individuals’ self-reported ability to appraise, express, regulate, and utilize their emotions enhanced their self-reported competence in managing interpersonal interaction. This heightened interpersonal competence mitigated students’ anxiety, subsequently enriching their satisfaction with life during the university lockdown. The non-significant direct effect of trait EI on anxiety, coupled with the significant relationship among trait EI, interpersonal competence, and anxiety, suggested that the students might have experienced temporary or situational anxiety caused by interpersonal competence during the university lockdown. This result further indicated that trait EI may not solely affect interpersonal relationships through social anxiety [67], but also influences general anxiety through interpersonal competencies, which eventually influence individuals’ satisfaction with life.

### 4.4. Practical Implication

The study’s results highlight the significant chain mediating effect of interpersonal competence and anxiety on the relationship between EI and life satisfaction. In addition, the results from the present study indicated a non-significant direct effect of EI on anxiety, and thus the non-significant meditating effect of anxiety on the relationship between EI and life satisfaction. This suggests that students’ anxiety might majorly stem from interpersonal interactions, which were significantly influenced by EI and, in turn, impacted their self-reported life satisfaction during the university lockdown. The findings imply that while EI may not directly alleviate general anxiety, it plays a crucial role in shaping students’ experiences and satisfaction with life through its effects on their social interactions.

For educators and staff within schools, it might be crucial to assess, nurture, and prioritize the development of students’ EI and interpersonal skills, particularly for those facing challenges or going through tough times. School psychologists, counselors, and social workers [97] are encouraged to enhance students’ capacities to evaluate, express, regulate, and utilize their emotions effectively. Cultivating EI may significantly contribute to building robust interpersonal competencies, which in turn may lead to increased social support. This support might be instrumental in reducing anxiety and boosting life satisfaction, ultimately equipping students with the resilience needed to navigate through difficult situations. By focusing on EI, schools can foster an environment that supports students’ overall well-being and success, both academically and personally. Concurrently, it is imperative for educators and school personnel to exemplify adept emotional regulation and possess a profound understanding of emotional knowledge. Demonstrating these emotional skills and knowledge has been correlated with the enhancement of teacher–student relationships and the facilitation of academic achievement, an effect that is observable even during the early stages of childhood development [98].

For individuals grappling with interpersonal relationships and anxiety, engaging in EI-focused practice activities, such as PATHS [99], INSIGHTS [100], and RULER [101], is highly recommended. However, two important considerations must be taken into account. First, some practices or interventions are designed as school-wide initiatives and may not be suitable for individual practice. Second, interpersonal and social skills are culturally specific. Thus, what is considered “appropriate” in terms of social and emotional conduct can differ based on the context and adheres to cultural norms [102,103]. It is essential to recognize and respect these cultural nuances when applying EI practices to ensure they are both effective and respectful of diverse backgrounds.

### 4.5. Limitations and Future Research

The present study has limitations. First, its findings rely solely on self-reported data. Even though performance-based assessments of EI may have greater predictive validity than self-reported EI [31], the conceptual importance of self-reported EI in subjective assessments was proposed by previous researchers [67], thus suggesting an area for future research on providing more empirical evidence for the predictive validity of self-reported EI, especially when studies involve emotional psychological assessments (i.e., anxiety and depression). One criticism of the use of self-reported measurement of EI, such as the one used in the current study, is that self-reported EI may be tapping other more well-established personality traits [31,104]. Upon visual scrutiny of the measurements utilized in this study, there is no definitive indication that the pronounced interconnectivity observed among EI, interpersonal competence, anxiety, and life satisfaction stems from similarities in the content of the items assessed. Secondly, the scope of the present study was confined to the examination of cross-sectional data. Although the study involved multiple variables and delved into the sequential mediating effects among EI, interpersonal competence, anxiety, and life satisfaction, the results were confined to a specific point in time. This suggests that a longitudinal research approach would be beneficial to investigate the enduring impact of EI on life satisfaction, mediated by interpersonal competence and anxiety, over an extended period. Thirdly, the findings of the present study were indicative of relationships during a specific timeframe, namely the early phase of the COVID-19 pandemic when universities were locked down. Even though the current study may capture individuals’ status related to the studied variables at this specific moment in time, it would be valuable for future research to re-examine these mediating effects under normal circumstances to assess the broader applicability of the results.

## 5. Conclusions

The findings from the current study offered empirical support for the mediating role of interpersonal competence between emotional intelligence and life satisfaction. They suggest that emotional intelligence plays a pivotal role in enhancing interpersonal skills, which subsequently have a positive impact on students’ life satisfaction. Additionally, the study uncovered a significant chain of mediation involving interpersonal competence and anxiety in the link between emotional intelligence and life satisfaction. This chain mediation further elucidates the internal influencing mechanism, demonstrating how emotional intelligence can affect students’ life satisfaction through its impact on interpersonal competence and anxiety, especially during the university lockdown. During this challenging time, the heightened frequency and intensity of interpersonal interactions emphasizes the crucial role of emotional intelligence and interpersonal competence in mitigating students’ overall anxiety and enhancing their overall life satisfaction. This could ultimately assist them in navigating through difficult circumstances.

## Figures and Tables

**Table 1:** Descriptive statistics and correlations of the main variables.

	M (SD)	1	2	3	4	5	6
Gender	1.76 (0.43)	-					
2. Age	21.29 (3.31)	-	-				
3. Emotional intelligence	2.94 (0.32)	−0.07	0.09	-			
4. Life satisfaction	3.99 (0.97)	0.00	0.07	0.47 ***	-		
5. Anxiety	1.70 (0.75)	0.07	−0.08	−0.24 ***	−0.41 ***	-	
6. Interpersonal competence	3.35 (0.60)	−0.15 ***	0.06	0.69 ***	0.51 ***	−0.39 ***	-

Notes. *n* = 297; gender: male = 1, female = 2; *** *p* < 0.001.

**Table 2:** Regression coefficients, standard errors, and model summary information of key variables.

Consequent									
	IC			Anx.			LS		
Variables	Coeff.	*SE*	*p*	Coeff.	*SE*	*p*	Coeff.	*SE*	*p*
EI	0.68	0.08	*p* < 0.001	0.05	0.17	0.544	0.23	0.19	*p* < 0.001
IC	-	-	-	−0.41	0.09	*p* < 0.001	0.26	0.22	*p* < 0.001
Anx.	-	-	-	-	-	-	−0.26	0.07	*p* < 0.001
Gender	−0.11	0.06	0.013	0.02	0.09	0.784	0.07	0.12	0.125
Age	0.01	0.01	0.882	−0.05	0.01	0.327	0.01	0.01	0.786
	*R*^2^ = 0.48 *F* (3, 293) = 90.82, *p* < 0.001			*R*^2^ = 0.15 *F* (4, 292) = 13.25, *p* < 0.001			*R*^2^ = 0.35 *F* (5, 291) = 31.26, *p* < 0.001		

Note: standardized coefficients are presented. EI = trait emotional intelligence; IC = interpersonal competence; Anx. = anxiety; LS = life satisfaction.

**Table 3:** Chain mediating effects of interpersonal competence and anxiety on the relationship between emotional intelligence and life satisfaction (bootstrap estimation).

Type of Paths	Pathways	Effect	95% CI	
			LL CI	UL CI
Direct Effect	EI → LS	0.23	0.00	0.32
Indirect Effect	EI → IC → LS	0.18	0.07	0.31
	EI → Anx. → LS	−0.01	−0.07	0.05
	EI → IC → Anx. → LS	0.07	0.03	0.12
Total Indirect Effect		0.24	0.09	0.40
Total Effect		0.47	1.12	1.75

Note: effect value = completely standardized effect. LL = lower limit; UL = upper limit; CI = confidence interval; EI = trait emotional intelligence; IC = interpersonal competence; Anx. = anxiety; LS = life satisfaction.

## Data Availability

The data utilized in this study can be made available upon receipt of a formal request to the corresponding author, in accordance with the established guidelines for data dissemination and intellectual property considerations.
